# Insights into Host–Pathogen Interactions in Biofilm-Infected Wounds Reveal Possibilities for New Treatment Strategies

**DOI:** 10.3390/antibiotics9070396

**Published:** 2020-07-10

**Authors:** Hannah Trøstrup, Anne Sofie Boe Laulund, Claus Moser

**Affiliations:** 1 Department of Plastic Surgery and Breast Surgery, Zealand University Hospital, 4000 Roskilde, Denmark; 2 Department of Clinical Microbiology, Copenhagen University Hospital, 2200 Copenhagen, Denmark; annesofielaulund@gmail.com (A.S.B.L.); moser@dadlnet.dk (C.M.)

**Keywords:** biofilm, chronic wounds, host response, S100A8/A9

## Abstract

Normal wound healing occurs in three phases—the inflammatory, the proliferative, and the remodeling phase. Chronic wounds are, for unknown reasons, arrested in the inflammatory phase. Bacterial biofilms may cause chronicity by arresting healing in the inflammatory state by mechanisms not fully understood. *Pseudomonas aeruginosa*, a common wound pathogen with remarkable abilities in avoiding host defense and developing microbial resistance by biofilm formation, is detrimental to wound healing in clinical studies. The host response towards *P. aeruginosa* biofilm-infection in chronic wounds and impact on wound healing is discussed and compared to our own results in a chronic murine wound model. The impact of *P. aeruginosa* biofilms can be described by determining alterations in the inflammatory response, growth factor profile, and count of leukocytes in blood. *P. aeruginosa* biofilms are capable of reducing the host response to the infection, despite a continuously sustained inflammatory reaction and resulting local tissue damage. A recent observation of in vivo synergism between immunomodulatory and antimicrobial S100A8/A9 and ciprofloxacin suggests its possible future therapeutic potential.

## 1. Introduction

Normal wound healing is a complicated, tightly regulated process in which a proliferative phase succeeds inflammation. The inflammatory phase lasts for approximately 48 h and is characterized by the influx of polymorphonuclear leucocytes (PMNs) and macrophages to the wound bed. The phagocytosis carried out by these immune cells prevents bacterial infection. This phase is followed by angiogenesis and the secretion of growth factors by fibroblasts and macrophages and, subsequently, the formation of a provisional extracellular matrix in a time span of approximately one week. Tissue remodeling will occur for the next one month to a year [1].

A chronic wound is one which fails to heal spontaneously within three months [2]. The reasons for the recalcitrance of some wounds are unclear, but emerging evidence point to a key role of *Pseudomonas aeruginosa* biofilm causing low-grade infection locally and causing a prolonged inflammatory state [3].

Predisposing factors for developing wound chronicity include chronic or acute infections, age, venous or arterial insufficiency, diabetes, neuropathy, renal impairment, malignancy, lymphedema, trauma, rheumatologic or other autoimmune conditions, pressure over a prominent bone, and immune suppression [4]. Venous leg ulcers, the most common type of non-healing ulcers, constitute 50–70% of all chronic ulcers and are caused by increased hydrostatic pressure due to venous insufficiency. *P. aeruginosa* infection is found in approximately 50% of venous leg ulcers. Although predominant in chronic venous ulcers, *P. aeruginosa* also challenges additional ulcers [5,6]. In the so-called post antibiotic era, *P. aeruginosa* is one of the predominant pathogens involved in burn wound infections, as these wounds are rapidly colonized after the skin’s natural barrier is damaged by thermal injury [7].

Clinical studies are often blurred by the heterogeneity of the etiology of wounds enrolled. Other studies may be limited due to the small number of wounds assessed. Comparing chronic venous leg ulcer fluids to acute wounds fluids, divergent results were found depending on the type of control wound chosen [8]. Superficial venous insufficiency can be managed by surgical intervention or split skin transplant in the case of non-healing wounds, although this is often challenged by *P. aeruginosa* biofilm infection [9]. The use of topical growth factors such as platelet-derived growth factor (PDGF) or allografts which release growth factors or the promotion of angiogenesis might be beneficial to healing in diabetic patients [10]. Similar results may be obtained by the wound application of autologous patches, constituting of leucocytes, thrombocytes, and fibrin generated from the patient’s own blood by special centrifugation [11]. Such application was shown to improve the healing of hard-to-heal diabetic ulcers as compared to the standard of care [12], and gives hope for new treatment approaches. However, the lack of basic knowledge of the causes of wound chronicity hinders truly successful medical treatment and ultimately worsens the prognosis for these patients. Chronic wounds can lead to devastating situations such as amputations and even death due to sepsis. There is a silent epidemic of biofilm-infected wounds, and now is the time for the thorough investigation of the underlying pathophysiological mechanisms of wound chronicity [13].

Translational medicine based on in vitro experiments and, more importantly, representative in vivo models can be used in the search for insights into the pathophysiology of host/pathogen interactions in chronic biofilm-infected wounds. Although Kadam and colleagues in 2019 showed that the number of publications concerning chronic wounds overall were increasing, a substantial paucity remains in publications on the basic science regarding the chronic wound microenvironment—i.e., on development of suitable laboratory model systems [14].

In the present review, we therefore discuss the impact of *P. aeruginosa* biofilm on the local and systemic host response from clinical and animal experimental observations and the current literature. Observations in mouse models will be considered for this review. With this background, implications for clinical wound healing are discussed and, finally, whether immunomodulatory topical treatment with the antimicrobial peptide, S100A8/A9, could be an adjuvant therapy strategy in chronic biofilm infections is also discussed.

## 2. Host/Pathogen Interactions in Chronic Wounds and Implications for Wound Healing

### 2.1. Bacteriology

Normal wound healing occurs in three phases—the inflammatory, the proliferative, and the remodeling phase. Chronic wounds are arrested in the inflammatory phase [15]. The immunogenicity of bacterial biofilms may be the explanation for stalled wound healing. Multiple bacterial species reside in the chronic wound environment. Most common are *Staphylococcus aureus*, the *Enterococcus* species, and *P. aeruginosa* [16,17]. *P. aeruginosa* is an opportunistic Gram-negative rod. The biofilm mode of growth is well described for this bacterium [18]. Bacterial subpopulations in the biofilms are metabolically less active. Adaptive and intrinsic mechanisms, such as the production of enzymes which are able to inactivate some antibiotics or by modifying cell permeability through efflux pumps, cause a thousand-fold resistance compared to planktonic bacteria, all in order to secure microbial survival [19].

A quantitative analysis of the cellular response towards biofilms in chronic wounds revealed that *P. aeruginosa* attracts more PMNs than *S. aureus* [20], which makes it an excellent choice for the assessment of host/pathogen interaction. *P. aeruginosa* biofilms are in human chronic wounds located in the subcutaneous fatty tissue [5,6], preventing their detection by standard wound swabbing techniques.

The majority of studies investigating the mechanisms of *P. aeruginosa* to avoid clearance and progress to a chronic infection are from patients with cystic fibrosis (CF) and lung infections. In particular, the regular sampling of CF sputum has enabled such studies which, to our best knowledge, have not been performed with chronic wounds [21]. The relatively high intrinsic antibiotic resistance and ubiquitous nature of *P. aeruginosa* as well as its well-known ability to develop further antibiotic resistance mechanisms is an important cause for the colonization of the wounds and the lack of standard antibiotic effect.

In the development of chronic infections, *P. aeruginosa* circumvents the early host defense by mechanisms that are not understood. Several metabolic changes—not virulence factors per se—of *P. aeruginosa* also occurs in establishing the chronic infection. One mechanism is by the secretion of elastase, which digests human thrombin and ultimately succeeds in preventing Toll-like receptor dimerization, thus avoiding host response [22]. Other ways to attenuate the host response is by rhamnolipid production, thereby impairing calcium-regulated pathways and protein kinase C activation [23], and complement inhibition by biofilm matrix exopolysaccharides [24] and LPS (smooth colony types) [25]. The Type III secretion system is another way to impair the innate immune response [26].

Furthermore, it has been described how *P. aeruginosa* transcription factors repress flagellar and pili genes and stress response regulator genes [27]. Especially flagellas can be strong stimulators of immune responses and have been used as a vaccine candidate [28]. The increased production of extracellular polysaccharides will be mentioned below. It has also been suggested that the well-developed ability of *P. aeruginosa* to adapt and generate subclones results in an insurance effect of the population and thereby the infection [29].

### 2.2. Experimental Models of Chronic Pseudomonas aeruginosa Biofilm Wound Infections

Experimental models comprise in vitro systems—e.g., cell cultures and in vivo animal models. Of the two, animal models are closer to the human wounds, as complex interactions between the host response and pathogens are present. Furthermore, in vivo models allow for clinically relevant endpoints. Rodents are the most commonly used in these settings. The use of larger animals such as pigs is often stated as providing a more human-like skin [30]. In contrast to the rodent’s dense layer of hair, thin dermis, and panniculus carnosus, pigs have a thick epidermis, well-developed rete-ridges, dermal papillary bodies, abundant subdermal adipose tissue, and similar dermal collagen and orientation and distribution of blood vessels in the dermis [31]. Rodents have an obvious wound contraction as wound closure, whereas pigs have a human-like healing dominated by epithelialization and similar turnover time [32]. Pigs can also encompass several wounds, numerous different topical treatments or infectious challenges, and have biopsies taken [33]. Despite these advantages, pigs have heterogenic skin anatomy, the costs are high, and need for space is challenging. Furthermore, it is extremely challenging to avoid the unintended colonization of the pigs’ wounds, and there exist only sparse reagents for the evaluation of host responses [34]. Still, rodents reveal several similar healing mechanisms to humans and are the most used species in wound research, albeit with different approaches and set ups [35,36,37]. Wounds are often incisional full thickness or burn wounds, although several additional strategies to generate the necessary initial skin defect have been described [38,39,40].

**Figure 1:**
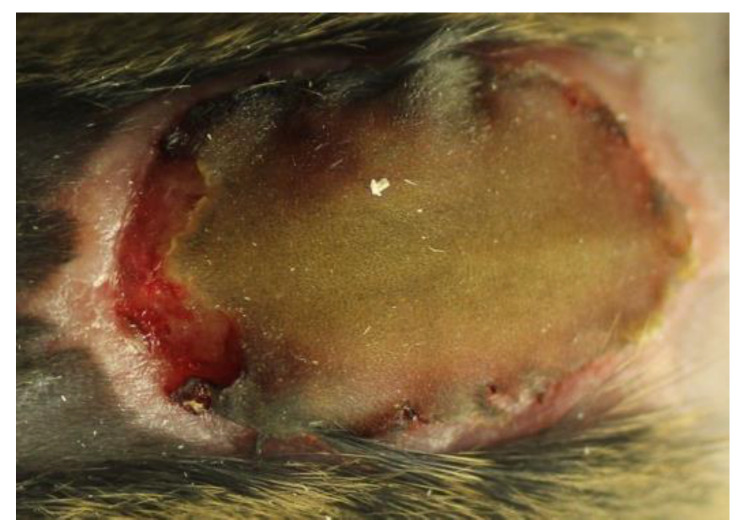
The appearance of an infected burn wound on a C3H/HeN mouse in the proliferative state of healing, approximately 7 days after infection (data not published). Note the peripheral healing red compartment and the central necrotic compartment.

The host–pathogen interplay in a chronic wound environment in the presence of *P. aeruginosa* biofilm infection can be assessed in a representative murine wound model. This model described below will be used for comparison throughout this paper. A pre-formed biofilm with alginate-embedded *P. aeruginosa* was injected subcutaneously beneath a full-thickness burn wound [41] on the back of anesthetized, shaved, immunologically diverse inbred strains of mice to explore the local and systemic host response [42]. Immediately post injury, the wounds are brown and homogenous. At day 2–3, they progress to resolution by peripheral detachment and the appearance of a red, vascularized healing area (Figure 1). The peripheral healing area can be compared to the necrotic central area of the wound.

The advantages of this model as compared to other established mouse wound models are the presence of a refractory, full-thickness skin necrosis and a long-lasting, exclusively local biofilm infection, with no systemic dissemination of bacteria. A shortcoming is that murine skin heals primarily by contraction, which could be circumvented by the use of splinting (see below, Section 3).

### 2.3. Host Response to Pseudomonas aeruginosa Biofilm Wound Infection

The mouse model has been successful for research groups studying chronic wounds and biofilms. For instance, the impact of the course of infection and the impact of biofilm on the bacteriology, histopathology, local and systemic host response, and consequences for wound healing in two different inbred mouse strains have been assessed in one strain susceptible to *P. aeruginosa* biofilm infection (BALB/c) and one relatively resistant (C3H/HeN) in a chronic *P. aeruginosa* lung infection model [43] and in a chronic wound model [42]. The BALB/c mouse strain with an established chronic *P. aeruginosa* biofilm infection is considered to be most representative of a chronic venous leg ulcer due to the increased lack of infectious control, in addition to an arrested healing process (see below). Using those two mouse strains, it was possible to evaluate the improved outcome of novel treatment strategies in the relatively susceptible BALB/c mouse strain. Accordingly, it was possible to investigate the factors suspected to be important for the aggravated course of the biofilm infection using the relatively resistant C3H/HeN mouse strain.

The host response towards *P. aeruginosa* wound infection can be characterized in a wound model by the following approaches:

1. Quantitative bacteriology and visualization of the bacteria and inflammatory cells in close proximity to the biofilm, located in the hypodermis.
2. Proinflammatory cytokines and neutrophil chemoattractant profiling.
3. Alterations in the growth factor profile in the proliferative state of healing.
4. The impact of the biofilm-mediated recruitment of PMNs from the bone marrow to the blood.

Using the setup with the two inbred mouse strains, we showed how the host factor profile is correlated to the outcome of *P. aeruginosa* biofilm infection in a chronic wound. In the natural course of infection, a lack of infection resolution in the susceptible mouse strain, BALB/C, was observed as compared to the resistant strain, C3H/HeN. The BALB/c mice had more *P. aeruginosa* biofilm in their wounds than the C3H/HeN mice at day four. The biofilms were in all samples located in the hypodermis of the skin [42]. No clearance of biofilm infection was observed.

Important differences in the chronic *P. aeruginosa* biofilm infection in the two mouse strains are provided in Table 1.

**Figure 2:**
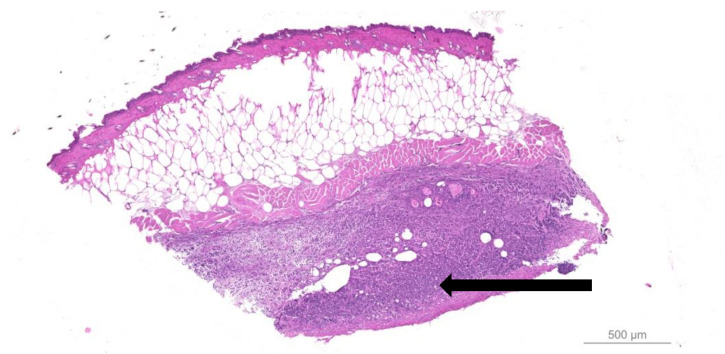
A representative hematoxylin and eosin-stained slide of a *Pseudomonas aeruginosa* biofilm-infected murine wound 7 days after infection (data not published). Note the darker purple inflammatory infiltrate containing leucocytes just below the panniculus carnosus in the hypodermis (black arrow).

Infected wounds display an inflammatory influx of PMNs and mononuclear cells (MNs) in response to bacterial infection [41]. Interestingly, the *P. aeruginosa* biofilm arrested BALB/c wounds in an acute, PMN-dominated inflammatory state [42], which is also described in human wounds [3]. An inflammatory infiltrate adjacent to the biofilms is visualized in the hypodermis of infected wounds (Figure 2).

### 2.4. Perturbation of Local Proinflammatory Cytokine and Growth Factor Profile by Pseudomonas aeruginosa Biofilm

The *P. aeruginosa* biofilm infection initially elicits a marked host response in wounds due to the virulence of *P. aeruginosa*. Virulence factors include lipopolysaccharide (LPS) and the most important exopolysaccharide, alginate, and protease production [44]. It has been suggested that *P. aeruginosa* secretes factors to dampen the local host response [23,26,45,46]. Accordingly, we observed and reported how the *P. aeruginosa* biofilm infection suppresses local neutrophil markers (S100A8/A9 and chemoattractants such as Keratinocyte-derived chemokine (KC) and the PMN mobilizer Granulocyte-colony stimulating factor (G-CSF)) in murine wounds, especially in susceptible mice. This induces a steady state, which may impair wound healing in a chronic course [47]. The local suppression of PMN-related markers in the presence of *P. aeruginosa* biofilm could be ascribed to the induction of a rapid necrotic killing of PMNs produced by these bacteria [48]. These observations are in accordance with our own research on suppressed S100A8/A9 in non-healing human wounds (a topic that is further discussed in a following section).

Furthermore, BALB/c mice are representative of a chronic wound model, as their wounds are arrested in the inflammatory phase of wound healing, expressing continuously high levels of Interleukin-1β (IL-1β) besides the lack of ability to gain infection control [42]. IL-1β, expressed by monocytes, is used as a marker for general inflammation caused by infection [49].

Growth factors essential in wound healing are vascular endothelial growth factor (VEGF), PDGF, and fibroblast growth factor (FGF), which are secreted by fibroblasts and macrophages in the proliferative phase of wound healing [50]. The wound growth factor profile is altered by the *P. aeruginosa* biofilm and correlated to the wound compartment and to the host defense profile [51]. VEGF is an angiogenic factor induced in human macrophages in hypoxic tissue or other situations of cell stress [52]. Being an endothelial cell mitogen, it contributes to neovascularization during wound healing. Furthermore, it has proinflammatory potential. It perpetuates local inflammation by PMN recruitment due to increasing vascular permeability [53]. The suppression of VEGF in necrotic tissue observed centrally in infected murine wounds could imply a reason for the topical growth factor substitution of this protein in clinical wound research. However, the proteolytic degradation of VEGF has been reported [54]. This may be the reason for the lack of clinical success in the use of topical growth factors in chronic wounds, since the prevalent proteolytic activity of the wound bed would presumably degrade the topically applied VEGF [55].

**Figure 3:**
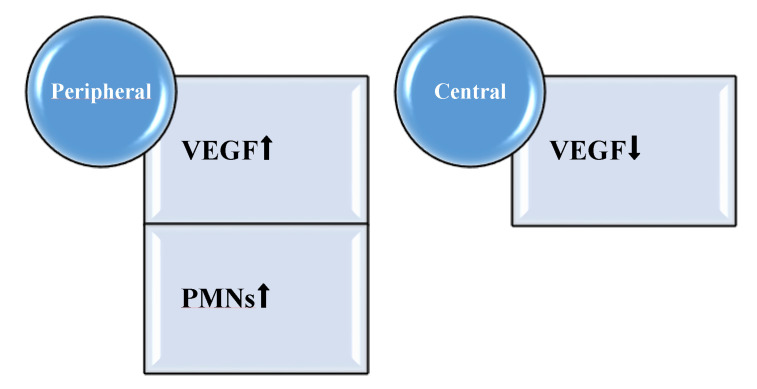
*Pseudomonas aeruginosa* biofilm increases vascular endothelial growth factor (VEGF) in the peripheral, healing compartments of the wounds, which also contain more polymorphonuclear cells as compared to the central compartment. Centrally, VEGF is suppressed.

Excessive amounts of VEGF act as a chemoattractant to *P. aeruginosa*, thereby exacerbating the infection [56]. Thus, the levels of VEGF were assessed using the chronic wound model in a setup where the host factor production was estimated and compared in the peripheral, healing part of the wound to the central non-healing and necrotic part of the wound. The *P. aeruginosa* biofilm infection induced VEGF protein levels by a factor 3 to 4 in the healing peripheral parts of murine wounds (Figure 3), thereby establishing the infection [51]. In a clinical study, we described a positive correlation between the levels of lipopolysaccharide and VEGF protein levels in wound fluids [57]. The expression of VEGF is strongly stimulated by tissue hypoxia, which is observed in the infectious environment caused by the oxygen consumption by the PMNs in respiratory bursts as well as the aerobic respiration of *P. aeruginosa* [58,59]. The insufficient supply of oxygen impairs wound healing and is the rationale for the therapeutic use of hyperbaric oxygen [60]. Furthermore, hypoxia impairs keratinocyte migration and proliferation in human chronic venous leg ulcers [61].

### 2.5. Systemic Impact of Pseudomonas aeruginosa Biofilm Infection in Animal Models of Chronic Wounds

Whereas G-CSF is the most important mobilizer of PMNs from the bone marrow, KC is an important chemoattractant for the extravasation of PMNs from the blood to the wound bed [62]. KC remained elevated in serum from BALB/c mice [42]. This may be an expression of the host response trying to recruit further immunoactive leukocytes to the chronic biofilm infected wound. Regarding the cellular systemic reaction to the infection in the response to *P. aeruginosa* biofilm, more PMNs are mobilized in the blood of susceptible BALB/c mice as compared to C3H/HeN mice [42]. The findings support that C3H/HeN mice have a more efficient immune reaction to the infection than the BALB/c mice, as a faster reduction in leukocytes was observed in the peripheral blood from C3H/HeN mice.; the total white blood cell count (WBC) and PMN count in the blood decreases more rapidly in the C3H/HeN strain. Interestingly, Sroussi and colleagues reported an inhibitory effect of S100A8 and A9 on neutrophil migration in vitro [63]. This observation is in accordance with our finding that topical treatment with S100A8/A9 for 5 days dampened the PMN count in blood from infected BALB/c mice [64].

### 2.6. Impact of Pseudomonas aeruginosa Biofilm on Murine Wound Healing

*P. aeruginosa* biofilm affects the host response by causing delayed wound healing [47]. The perturbation of host response causes significant tissue damage to the skin, thereby hindering wound resolution. With the use of translational research—e.g., by animal chronic wound models—important factors for healing have been revealed. In clinically relevant animal models—e.g., Zhao et al. [65]—*P. aeruginosa* biofilm infection delayed wound healing in diabetic mice. Accordingly, Watters et al. demonstrated a lack of infection resolution and impaired wound healing by the infliction of full-thickness surgical excision wounds on diabetic mice backs, inoculated with *P. aeruginosa* PAO1 to each wound [66]. The fact that mice heal predominantly by contraction and less by the emergence of granulation tissue as in humans is addressed by Ahn and Mustoe, who developed a wound model using a rabbit ear. In this model, the underlying cartilage functions as a splint, thereby circumventing the wound contraction [67]. Although other models exist, it should be highlighted that mouse models can reveal important healing parameters.

Wound healing can be evaluated by the digital planimetric assessment [68] of the reduction in the total wound area, by the size of necrosis and subsequent analysis ImageJ^®^ [69,70,71]. Interestingly, infected susceptible BALB/c mice are delayed healers compared to C3H/HeN mice, since the C3H/HeN mouse strain reached a reduced wound size and a reduced area of the central necrosis as compared to the BALB/c mouse strain [47,51]. Actually, and in accordance with this, Li et al. described BALB/c genetically as slow healers compared to C3H/HeN mice, who were characterized as intermediate healers [72]. Those observations support the use of the BALB/c mouse strain as a model of chronic wounds and poor healing.

## 3. Topical Intervention on *Pseudomonas aeruginosa* Biofilm-Infected Wounds through Mouse Models

Evidence of clinically effective treatment modalities is surprisingly scarce. The chronic biofilm formation is an efficient mechanism against host response and antibiotic treatment [73]. Enzymes capable of degrading the biofilm are thus an interesting suggestion. Indeed, Fleming and Rumbaugh recently showed how glycoside hydrolases disperse biofilms. In addition, they augmented the efficacy of Meropenem towards *P. aeruginosa* infection in a full thickness murine wound model [74]. Further studies of the clinical implications are awaited.

Another approach describes the advantages of naturally occurring antimicrobial peptides (AMPs), which protect the skin from bacterial invasion [75]. In previous studies, it was shown that S100A8/A9 RNA and protein levels are upregulated in the epidermis of acute murine as well as human wounds [76]. Furthermore, chronic human wounds lack S100A8/A9 [57,77]. S100A8/A9, also known as Calprotectin, is a calcium-binding, heterodimeric protein, constituting 40–60% of the cytosol of PMNs and 5% of monocytes. It is an alarmin, a constitutively available endogenous molecule. It is released from dead or necrotic cells upon tissue damage [78]. In a non-healing wound setting, mesenchymal stem cells were subjected to human recombinant S100A8/A9 accelerated healing of murine full-thickness wounds [79]. In a paper on *P. aeruginosa*-induced keratitis, the authors discuss the role of S100A8/A9 in the host defense towards *P. aeruginosa* infections in vivo, as the genes for S100A8/A9 seem to be the most highly upregulated by bacterial flagellin exposure. Flagellin is a ligand of the Toll-like receptor which is able to recognize pathogen-associated molecular patterns to initiate immune responses. The upregulation of such genes results in a flagellin-induced protection, whereas the functional blocking of both peptides increased the susceptibility to *P. aeruginosa* infection [80]. In another model of murine fungal keratitis, 1 µg of recombinant S100A8/A9 injection into the corneas of S100A9^−^/^−^ mice restored the ability to inhibit the hyphal growth of *Aspergillus fumigatus* 24 h post infection [81]. Both those studies support the existence of the direct antimicrobial activity of S100A8/A9, at least in the planktonic state.

Topical recombinant S100A8/A9 injected underneath the wounds of *P. aeruginosa*-biofilm infected wounds in BALB/c mice ameliorated local wound infection after 5 days of treatment [64]. In a later study, S100A8/A9 augmented the effect of 1 mg of systemic ciprofloxacin in the same model of chronic biofilm-infected wounds in BALB/c mice [82]. When S100A8/A9 was combined with systemic ciprofloxacin, the bacterial load was lowered significantly, even after 3 days of therapy, and the levels of the individual S100A8 and S100A9 was further increased. Besides being a consequence of the topical therapy, the S100A8/A9 increase is interpreted as a surrogate marker of promoted wound healing. Interestingly, no in vitro synergistic effect between S100A8/A9 and ciprofloxacin was shown. This emphasizes that the synergistic ciprofloxacin potentiating effect of S100A8/A9 is highly dependent on host cells and further underlines the importance of using representative animal models, if it is not possible to proceed directly to clinical trials. The clinical implication in a non-healing wound setting of the synergistic S100A8/A9 effect is further stressed due to the relatively poor penetration of antibiotics to a skin focus, even with a biofilm infection—adding an antibiotic augmenting compound could potentially compensate for this phenomenon. The results presented directly point towards clinical testing and possible new therapeutic approaches. Whether the combination therapy of S100A8/A9 and ciprofloxacin will prevent the development of bacterial resistance and improve wound healing is currently being investigated.

S100A8/A9 may be released by active phagocytes, acting as a dose-dependent switch by initially stimulating phagocytosis, but in higher concentrations terminating neutrophil recruitment, acting more as an anti-inflammatory agent. Thus, one could speculate that the lack of S100A8/A9 is an expression of a chronic infection: The host defense is incapable of resolving the local infection, as PMNs are efficiently recruited from the blood to the site of infection but are counteracted by the close proximity to the biofilms. A major challenge in the use of S100A8/A9 as an adjuvant to ordinary antimicrobial treatment is the challenge of the dose-dependent effect, as the dual effects of alarmins are known [78]. The growth factor potential of these innate host response proteins depends on release, dose, and context as they may mediate repair after injury [76]. Human and animal model observations strongly indicate that the S100A8/A9 response is perturbed and inappropriate in chronic wounds. Further studies are warranted to describe the pathophysiological impact of the multifaceted role of S100A8/A9 on biofilm-infected wounds. Of course, the lack of permission to use S100A8/A9 clinically has to be solved, but promising results may promote a solution to this challenge.

## 4. Conclusions

**Figure 4:**
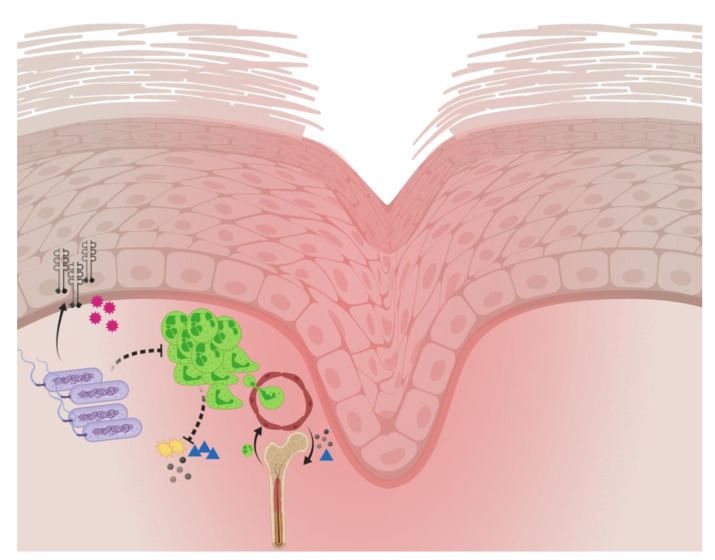
The hypothesis of the host/pathogen interaction between biofilm (Gram-negative rods in biofilm aggregates, left) and incoming polymorphonuclear neutrophils (green large cells, right). The biofilm causes increased levels of interleukin-1β (black dimers) and vascular endothelial growth factor in the wound periphery (pink spiky balls), and it inhibits neutrophil activity, reflected by reduced levels of S100A8/A9 (yellow stars), Keratinocyte-derived Chemokine (blue triangles), and Granulocyte-Colony Stimulating Factor (black dots). Full arrows: promotion; dotted arrows: inhibition. This figure was created with Biorender.com.

Chronic wounds are arrested in the inflammatory phase of wound healing for reasons that are unknown. Host/pathogen interaction is ultimately detrimental to wound healing. *P. aeruginosa* biofilm affects wound healing negatively via alterations in host defense mechanisms; PMNs are initially recruited to the site of infection. S100A8/A9, KC, and G–CSF are locally dampened by *P. aeruginosa* biofilm, which establishes chronicity. In the chronic state, the PMNs proceed into a quiescent phase, unable to resolve the infection. Loops of these distorted pathways cause high levels of local tissue damage and contribute further to wound development (see Figure 4). The wound environment is characterized by high proinflammatory IL-1β and proangiogenic VEGF, although the latter seems repressed by the biofilm or the central necrosis. Still, the inflammation is inappropriate and lacks the resolution of the infection due to the attenuation of phagocytic cell activity, despite the continuous recruitment of such cells. This may play a pivotal role in the modulation of the tissue repair response to infection by inducing premature cellular senescence [83].

The restoration of normal PMN activity holds a potential for the adjunctive therapy of wound chronicity. The impact of *P. aeruginosa* biofilm and the host defense interaction on wound fibroblasts and keratinocytes, crucial to wound healing, are also areas of great importance and deserve further investigation.

Overall, due to the risk of surgical intervention or even amputation, there is a need to assess the basic science of wound chronicity and rethink strategies to combat the biofilms in chronic wounds. In order to substantiate a role for the use of topical antimicrobial peptides or other local interventional therapies, biofilms should be taken into consideration. This includes regimes with sufficient antibiotic doses, combination antibiotic therapy targeting different niches of the biofilms, topical treatment being considered, and the use of antibiotics with a good penetration of the skin; prolonged or repeated treatments might be beneficial. In addition, correct sampling should be used—biopsies are preferable to swabs. The gold standard level of proof would be a randomized, controlled study in groups of patients with venous leg ulcers and arterial or diabetic ulcers. Confounding factors should be minimized, and patient homogeneity could ensure group compatibility and improve the internal strength of such clinical studies.

All the procedures performed in studies involving animals were in accordance with the ethical standards of the institution or practice at which the studies were conducted (approved by the Animal Ethics Committee of Denmark (2010/561-1766).

## Figures and Tables

**Table 1:** Host response to *Pseudomonas aeruginosa* biofilm wound infection in two immunologically diverse mouse strains.

C3H/HeN	BALB/c
Resistant towards infection Faster infection control	Susceptible to infection Aggravated inflammatory IL-1β response No infection control
Faster wound closure	Delayed wound closure
